# A chair at the table: a scoping review of the participation of refugees in community-based participatory research in healthcare

**DOI:** 10.1186/s12992-021-00756-7

**Published:** 2021-09-06

**Authors:** Tali Filler, Pardeep Kaur Benipal, Nazi Torabi, Ripudaman Singh Minhas

**Affiliations:** 1 Department of Pediatrics, St. Michael’s Hospital, Unity Health Toronto, Toronto, Canada; 2 Faculty of Medicine, University of Toronto, 61 Queen Street East, 2nd Floor, Toronto, ON M5C 2T2 Canada; 3 Library Services, St. Michael’s Hospital, Unity Health Toronto, Toronto, Canada; 4 Division of Developmental Pediatrics, Department of Pediatrics, University of Toronto, Toronto, Canada

**Keywords:** Refugee, Asylum-seeker, Community-based participatory research, Health intervention, Health policy

## Abstract

**Background:**

Refugees often face psychosocial complexity and multi-dimensional healthcare needs. Community-Based Participatory Research (CBPR) methods have been previously employed in designing health programs for refugee communities and in building strong research partnerships in refugee communities. However, the extent to which these communities are involved remains unknown.

**Objective:**

To review the evidence on the involvement of refugees in CBPR processes to inform healthcare research.

**Methods:**

A scoping review was performed, using Arksey & O’Malley’s methodological framework. A literature search in Medline, PubMed, PsycINFO, CINAHL, Embase, Global Health, Scopus, and Policy File Index for articles published until August 2020 was conducted. Articles were included if they focused on CBPR, had refugee involvement, and discussed healthcare/health policy.

**Results:**

4125 articles were identified in the database searches. After removal of duplicates, 2077 articles underwent title and abstract review by two authors, yielding an inter-reviewer kappa-statistic of 0.85. 14 studies were included in the final analysis. The purpose of CBPR use for 6 (42.9%) of the articles was developing and implementing mental health/social support interventions, 5 (35.7%) focused on sexual and reproductive health interventions, 1 (7.1%) focused on domestic violence interventions, 1 (7.1%) focused on cardiovascular disease prevention and 1 (7.1%) focused on parenting interventions. In terms of refugee involvement in the various stages in the research process, 9 (64.3%) articles reported refugees having a role in the inception of the research, no articles reported including refugees in obtaining funding, all articles included refugees in the design of the research study, 10 (71.4%) articles reported having refugees involved in community engagement/recruitment, 8 (57.1%) articles reported involvement throughout the data collection process, 4 (28.6%) articles reported involvement in data analysis, 6 (42.9%) articles reported having refugees involved in knowledge translation/dissemination and 1 article (7.1%) reported having refugees contribute to scale up initiatives.

**Conclusions:**

CBPR has been identified as a methodology with the potential to make substantial contributions to improving health and well-being in traditionally disenfranchised populations. As the needs of refugee communities are so diverse, efforts should be made to include refugees as partners in all stages of the research process.

**Supplementary Information:**

The online version contains supplementary material available at 10.1186/s12992-021-00756-7.

## Introduction

With evolving global conflicts, climate change and mass displacement of communities, the current refugee crisis is of urgent relevance to governments, healthcare providers and decision-makers [1, 2]. Upon arrival to their country of resettlement, families experience a number of challenges in accessing the support they require to fully integrate into their new community. Refugees also face disrupted social networks, economic instability, mental health concerns due to pre-migration exposure to personal or vicarious violence, language barriers, changes in cultural adaptation and resettlement, frequent relocation and migration, all of which can impact health, education and employment [3, 4].

In recent years, international calls have been made to prioritize addressing the institutional and structural injustices refugees face that contribute to poor health outcomes [5]. It is well understood that refugees often face psychosocial complexity and multi-dimensional healthcare needs, and current health systems perpetuate the ongoing health inequities faced by these communities. Health systems and services are often positioned so that those with precarious status are unable to access the care that they need, attempting to discourage the entry of new migrants [6]. Effectively addressing institutional and structural injustices involves reassessing the way healthcare is approached and restructuring the current systems to create action points to address the social determinants of health in an ethical manner [5].

Community-Based Participatory Research (CBPR) has been identified as a methodological approach with the potential to make substantial contributions to improving the health and well-being of traditionally disenfranchised populations while building capacity within these communities [7, 8]. CBPR engages community members as active and equal participants in every phase of the research process, facilitates trust-building, and enables the rigorous and ethical conduct of research with the refugee community. CBPR methods allow for tangible benefits for all partners involved. This includes the ability to gain a deeper understanding of complex community and institutional issues, and opportunities to enhance capacity-building amongst academic and community partners. CBPR approaches have been successfully employed to create interventions and programs that address the complex healthcare needs of refugee populations, in designing mental health programs for refugee children and adolescents, and in building strong research partnerships with refugee communities [7].

The core principles of CBPR state that all research using this methodology: 1) is participatory, 2) is cooperative, and creates partnerships that are collaborative and equitable, 3) is a cooperative learning process with a mutual exchange of expertise between all partners, 4) involves systems development and sustainability, and builds on the strengths of the community, 5) involves empowering all partners through mutual decision making, ownership of research and findings, and knowledge dissemination, 6) entails implementing an intervention based on the research, 7) recognizes the community as a social entity with a unique identity, and 8) requires a long-term commitment by all partners [9]. CBPR is typically an iterative process, by which community partners are involved in various stages of design and revision of the proposed intervention.

For CBPR to be implemented successfully, it should be present throughout the entire research process [10]. The research process has been defined as having a number of different stages, including the following: 1) inception of research problem/policy need, 2) obtaining funding, 3) study design, 4) engaging community/recruitment, 5) data collection, 6) data analysis, 7) knowledge translation and dissemination and 8) scale up [10]. As the needs of refugee communities are so diverse, efforts should be made to include refugees in all stages of the research process. Previous research has shown that in order for CBPR to be implemented most effectively, partners must be involved in all steps of the research process [11]. While CBPR methods have proven to be successful with refugee communities in general [7], the specific steps in which they participate in the CBPR process remains unknown. With the growing refugee crisis and associated healthcare needs, their involvement in the development of health interventions is essential. Therefore, this scoping review aims to review refugee involvement in the CBPR process related to healthcare and provide key recommendations on how to partner with this population in future research activities. The current study aims to answer the following research question; how have refugee communities been involved in contributing to CBPR healthcare research and design?

## Methods

### Data sources and search strategy

A search strategy was developed by an information specialist (N.T.) and three reviewers (P.K.B., T.F. and R.S.M.). A literature search in Medline (Ovid), PubMed, PsycINFO (Ovid), CINAHL (EBSCO Host), Embase (Ovid), Global Health (Ovid) Scopus, and Policy File Index (Proquest) for articles published until August 6, 2020 was conducted (Additional file 1). A combination of subject headings and text words were used for each of the main search concepts: community-based participatory research, refugees/asylum-seekers, health care and health policy. The search was limited to English-language articles only. Reviews and conference papers were removed. The Medline search was peer-reviewed by a second information specialist using the PRESS guideline and the feedback was incorporated prior to running the search in other databases [12].

### Data extraction and synthesis

The duplicated articles were removed using EndNote X9.2 software. An initial title, citation and abstract review was conducted by two reviewers (P.KB. and T.F.) according to the inclusion and exclusion criteria (Table 1). While there is regional variation in the specific definition of a refugee or asylum seeker, all individuals who were identified in studies as refugees, or those who “experienced well-founded fear of being persecuted for reasons of race, religion, ethnicity, nationality, membership of a particular social group, or political opinion,” [13] were included in this study. Both reviewers independently screened all articles and the third reviewer (R.S.M.) resolved discrepancies between primary reviewers. Data were organized, extracted and analyzed according to Arksey and O’Malley’s descriptive analytic model for scoping reviews by two authors (P.KB. and T.F.) [14]. Inter-rater reliability was assessed using kappa statistics between the first and second author. The study team reviewed disagreements and only included articles for full-text review that both reviewers agreed upon. Articles were excluded if they were not peer-reviewed, commentaries, or case reports, and did not describe the use of CBPR in refugee communities. Data extracted included ethno-racial data, country of origin, purpose of CBPR, CBPR principles used, and its influence on healthcare research and policy.

**Table 1:** Inclusion and Exclusion Criteria for Study Selection

Inclusion Criteria	Exclusion Criteria
Available in English Full-Text Available Focuses on Refugee or Asylum-Seeking Background Only Uses CBPR/PAR as methodological philosophy Discusses healthcare research or health policy Primary research articles, commentaries and case reports	Reviews, book chapters, dissertations, conferences or other abstracts for which a full text has not been published Does not specifically focus on refugee population (i.e., includes immigrants and does not distinguish between the two groups)

### Manuscript preparation and reporting

This study was conducted, and manuscript was written in accordance with the Preferred Reporting Items for Systematic reviews and Meta-Analyses extension for Scoping Reviews (PRISMA-ScR) Checklist and PRISMA-S Checklist [15, 16]. The Checklists are available in Additional files 2 and 3.

## Results

**Fig. 1:**
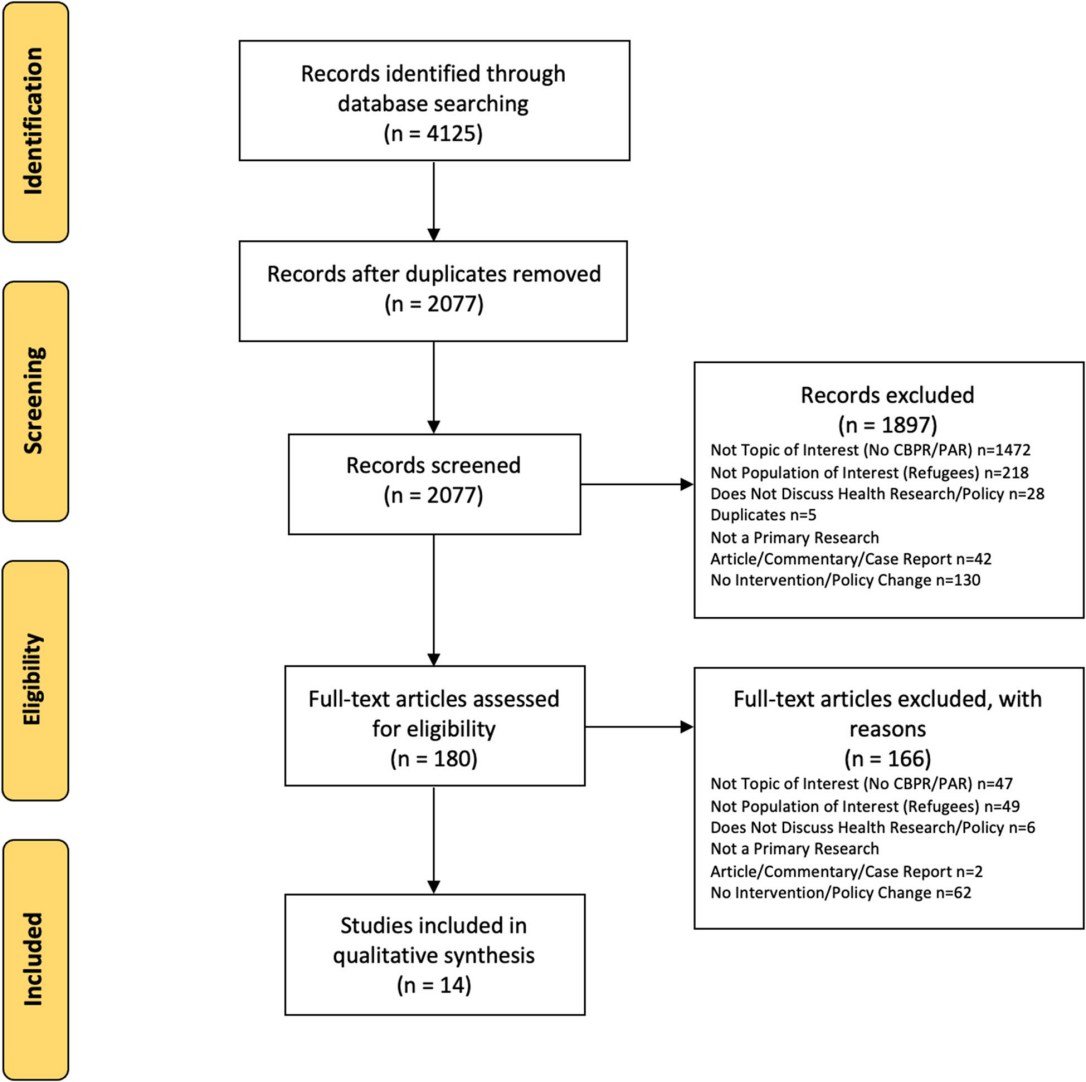
PRISMA Flow Diagram

The study team identified a total of 4125 articles in the database searches, as described in Fig. 1. After removal of duplicates, 2077 articles underwent title and abstract review by two authors, yielding an inter-rater kappa-statistic of 0.85, suggesting high agreement. 1897 articles were excluded based on title and abstract review. A total of 180 articles underwent full-text review. Among those screened, 62 articles were excluded as they did not discuss a healthcare intervention or health policy. 49 articles were excluded because they did not include the population of interest (refugees). Given the unique pre-migration, migration and post-migration experiences of refugees, studies were excluded if they conflated immigrants and refugees and did not differentiate their involvement or experiences. 47 articles were excluded as they did not focus on CBPR/PAR methodology. While CBPR and PAR are sometimes used interchangeably, they do have slight methodological differences, as CBPR emphasizes the involvement of the community as an entity, defining it as existing beyond the confines of the research project itself [17]. Therefore, articles that were labelled as PAR but incorporated CBPR methodology (i.e. emphasized community-level engagement) were included. 6 articles were excluded as they did not focus on healthcare research/policy. Finally, 2 articles were excluded as they were not a primary research article, commentary or case report. We did not identify additional items in the references of eligible studies. A total of 14 studies were included in the final analysis after meeting all inclusion criteria [18–31].

### Study characteristics

**Fig. 2:**
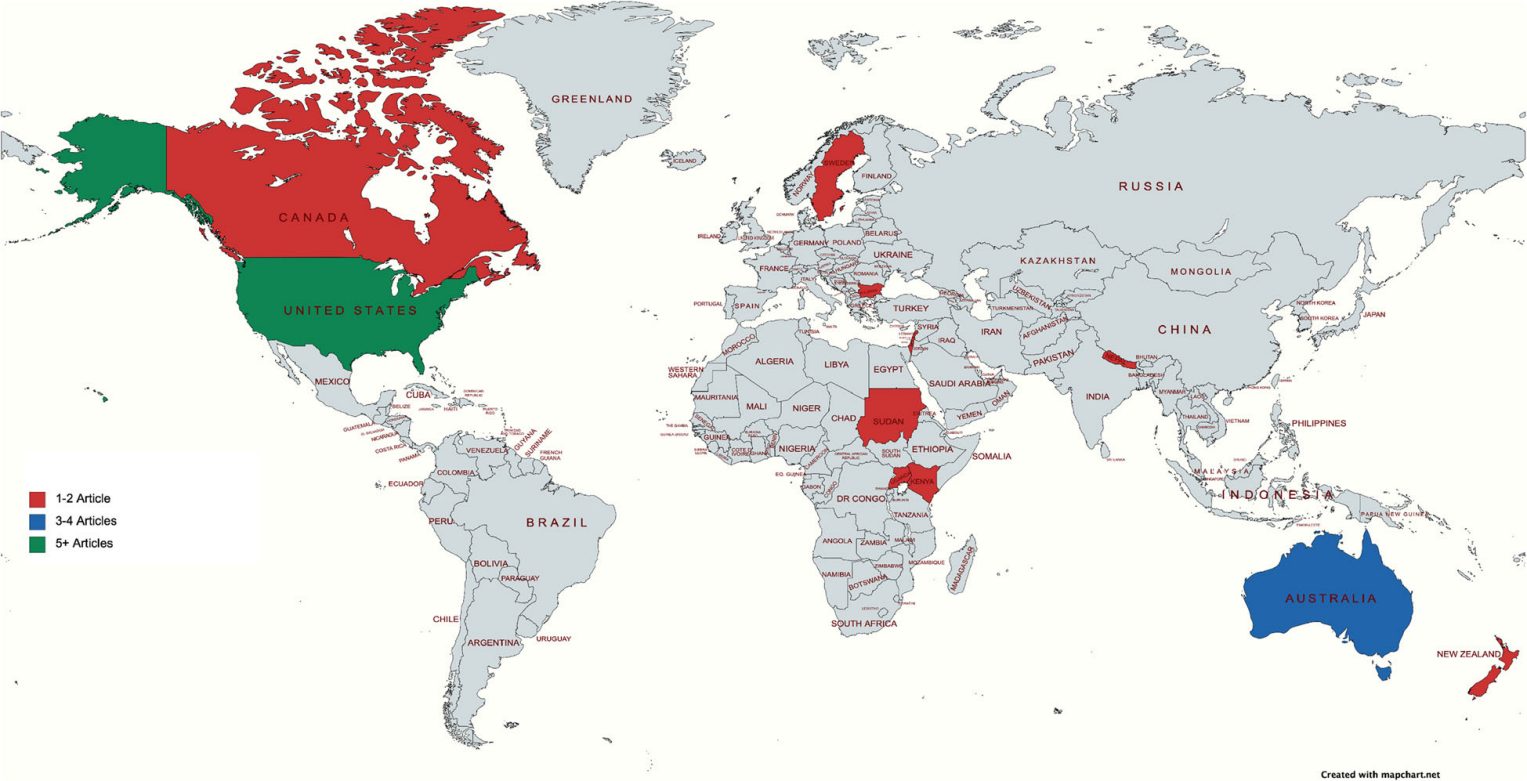
Geographic distribution of study location. Study locations included the United States, Australia, Canada, Lebanon, New Zealand, Palestine, South Sudan, Rwanda, Kenya, Nepal, Uganda and Sweden

Study location is summarized in Fig. 2 and study characteristics are summarized in Table 2. 6 of the 14 studies included were conducted in North America, 3 were conducted in Australia/New Zealand, 2 were conducted in the Middle East, 2 were conducted in Africa and 1 was conducted in Europe. Race and ethnicity data were included in 13 of the 14 studies (92.9%). The race/ethnicity of refugees included South Sudanese, Bhutanese, Somali Bantu, Somalian, Hmong, Great Lakes Region African, Iraqi, Cambodian, Ethiopian, Eritrean, Nigerian, Egyptian, Lebanese, Jordanian, Saudi Arabian, Syrian, Palestinian, Congolese, Karen and Mandaean. However, the country of origin of refugee participants was reported to a slightly lesser degree (10/14 studies, 71.4%). Those reported include Sudan, Somalia, Bhutan, Kenya, Nepal, US, India, Cambodia, Palestine, Rwanda, Burma and Iraq. In terms of age of study population, 10 studies (71.4%) focused on adult participants (over 18 years of age), 3 studies (21.4%) included participants of all ages and 1 study (7.14%) focused primarily on adolescent participants. Articles were published from 2006 to 2020, with an increase of studies seen from 2017 onwards.

**Table 2:** Study Characteristics of Included Studies

First Author Last Name	Year of Publication	Race & Ethnicity Data Captured (Yes/No)	Race & Ethnicity Data	Age of Study Population	Purpose of CBPR
Afifi	2011	Yes	Palestinian	Adolescents	Mental health
Baird	2015	Yes	South Sudanese	Adulthood	Women’s mental health, sexually transmitted infections, parenting
Betancourt	2020	Yes	Bhutanese, Somali Bantu	All Ages	Mental health
Goodkind	2017	Yes	Hmong, Great Lakes Region African, and Iraqi Refugees	Adulthood	Social support intervention
Grigg-Saito	2008	Yes	Cambodian	Adulthood	Cardiovascular health and diabetes
Guerin	2006	Yes	Africa (Somalia, Ethiopia, Eritrea, Sudan and Nigeria) and Middle East (Egypt, Lebanon, Iraq, Jordan, Saudi Arabia and Syria)	Adulthood	Reproductive health (female genital cutting advocacy)
Gustafson	2013	Yes	Sudanese	Adulthood	Domestic violence
Miller	2020	Yes	Palestinian Refugees	Adulthood	Parenting
Pavlish	2017	Yes	Congolese Refugees	Adulthood	Women’s health
Riggs	2017	Yes	Karen Refugees	Adulthood	Pregnancy and women’s health
Signorelli	2015	Yes	Karen and Mandaean	Adulthood	Mental health
Stewart	2011	Yes	Sudanese and Somali	Adulthood	Mental health
Tanabe	2018	Yes	Bhutanese	All Ages	Reproductive health and disability inclusion
Warner	2019	No	–	All Ages	Mental health

### Purpose of CBPR approach

The purpose of the health interventions targeted by the CBPR approach varied amongst studies. However, 2 themes were commonly addressed throughout a majority of the studies. Of the articles included, 6 (42.9%) focused on developing and implementing mental health/social support interventions, and 5 (35.7%) focused on sexual and reproductive health interventions. One article (7.1%) focused on domestic violence interventions, another (7.1%) focused on cardiovascular disease prevention and the last (7.1%) focused on parenting interventions. Only 2 (14.3%) of the 14 studies included noted compensation for refugees who partnered throughout the CBPR process. While we aimed to include studies that focused on either a) healthcare intervention or b) health policy, the studies identified in our search that included the participation of refugees as partners were only focused on healthcare intervention.

### Summary of refugee involvement and representation

**Fig. 3:**
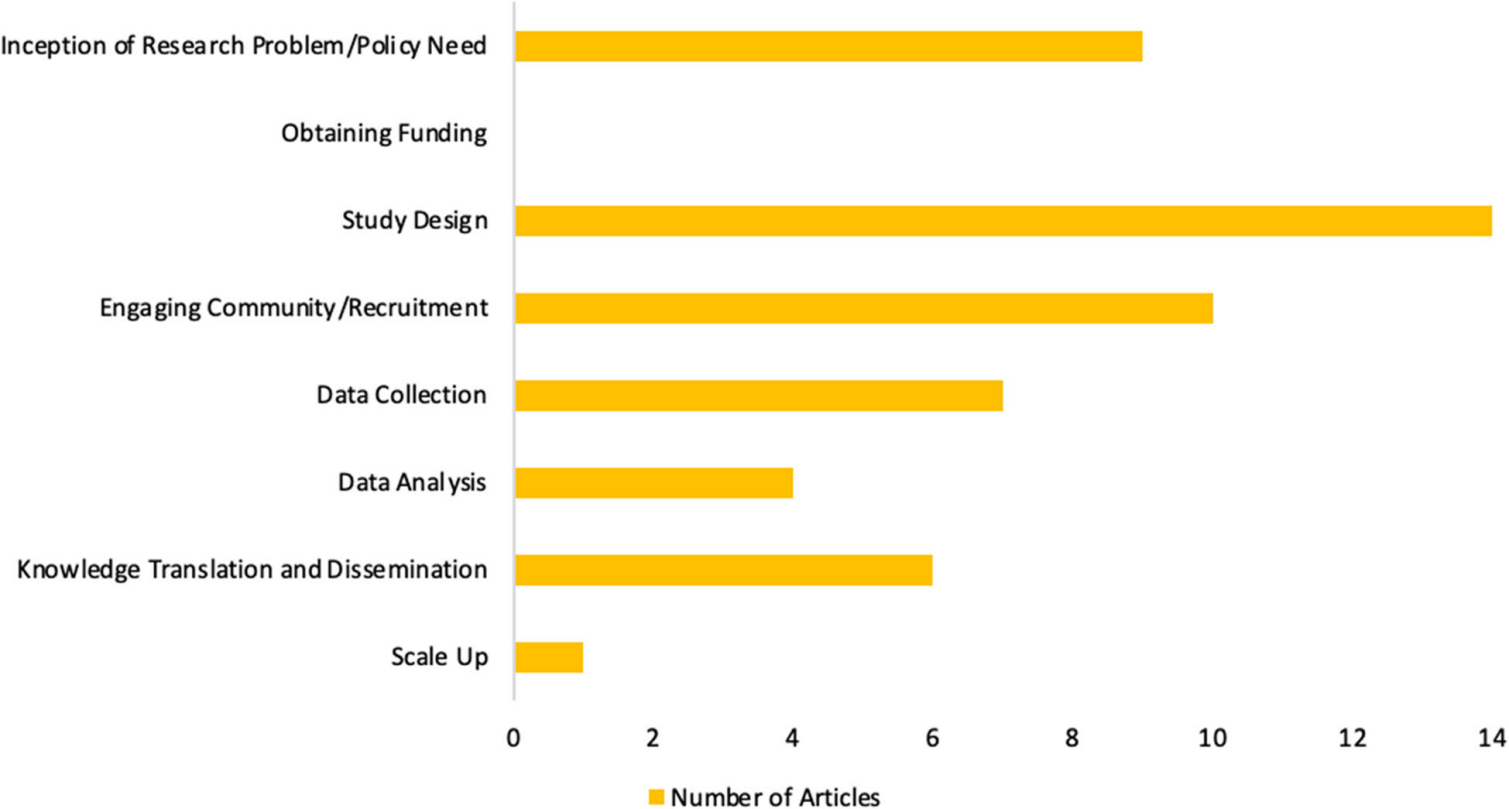
Refugee Involvement in CBPR Processes

The use of CBPR methods places an emphasis on the importance of having participants as partners throughout all stages of the research process. We identified 8 key steps that constitute the full research process, namely 1) inception of research/ policy need, 2) obtaining funding, 3) study design, 4) engaging community/recruitment, 5) data collection, 6) data analysis, 7) knowledge translation and 8) scale up. We identified trends in the involvement of refugees in certain steps of the research process that predominated over others in the included articles. Refugees were highly represented in step 1 of the research process, inception of research/policy need. Of the studies included, 9 (64.3%) reported refugees having an important role in this step. Step 3 was even more highly represented with refugee involvement, as all 14 articles reported including refugees in the design of the research study. Step 4, engaging community/recruitment, was widely reported by articles (10, 71.4%) as having significant refugee involvement. Step 5 was also reported but slightly less frequently, as 8 articles (57.1%) reported having refugee involvement throughout the data collection process. A summary of these results can be found in Fig. 3.

### Gaps in involvement of refugees

There were a number of areas throughout the research process where refugee involvement either did not take place or was not reported. Of particular note was step 2, obtaining funding, which was not reported in any of the articles as having refugee involvement (0%). Refugee involvement in data analysis, step 6, was only reported in 4 articles (28.6%). 6 articles (42.9%) reported having refugees involved in step 7, knowledge translation/dissemination, and 1 article (7.1%) reported having refugees partake in step 8, scale up.

## Discussion

Overall, studies noted the immense value of having refugee partners, which allowed their interventions to be more successful as they were rooted in the community. The unique insights refugees brought forth allowed study processes to be carried out more smoothly. For instance, the study by Goodkind et al. discussed the potential for refugee mistrust in their randomized control trial [21]. The community advisory council, which included refugee community partners, suggested implementing a public showing of the random assignment portion of their study so members of the community could directly observe that the selection was truly randomized. The insight from refugee community partners directly impacted the study design to improve its success and make it more appropriate for the local context [21].

Our results also demonstrate that despite the growing emphasis on community involvement and engagement in health research, there is a lack of refugee involvement in the full scope of the CBPR process, as well as in health policy design. Previous research has shown that the use of community partners helps successfully facilitate policy change [32, 33]. It is clear from this work that there is a current gap in collaboration with refugee partners in policy design and advocacy.

It is important to recognize that refugees are a diverse, heterogeneous group with varying profiles, strengths and challenges [7]. Refugees come from different countries, cultural and religious backgrounds, varying races and ethnicities that all contribute to their pre-migration, migration and post-migration experiences, which thereby influences their health outcomes to varying degrees [8]. As part of the iterative nature of CBPR methodology, the process should be conducted with each unique group until their needs are met. Our results demonstrate that literature examining refugee involvement in CBPR conflated race and ethnicity variables, as well as the age of participants. Previous research has shown that race and ethnicity should be captured separately, as there are different meanings ascribed to the terms [34]. While there is little consensus on concrete definitions of race and ethnicity, they are both socially constructed concepts that may represent different aspects of identity. Often, race is used to describe shared physical traits as well as cultural patterns. Ethnicity is often used to describe shared cultural practices, beliefs, traditions and language [35]. Johnston-Guerrero suggested that race is more often determined externally, whereas ethnicity is more often determined internally [36]. As such, these concepts may differently impact one’s context, experiences and identity. Therefore, reporting these terms separately in research is critical to ensure experiences amongst various refugee groups are not conflated.

Results also showed the lack of involvement of refugees in specific stages of the CBPR process, with the lowest involvement in obtaining funding and scale up opportunities. Barriers to full participation in research processes should be examined further in order to eliminate health disparities and further build capacity amongst refugee communities.

Issues surrounding trust were discussed in a number of the included studies, which is consistent with previous research findings. The interactions between researchers and research participants have not always been mutually exclusive, which has created a sense of skepticism and mistrust amongst participants who may feel disadvantaged [37]. Historically, research participants have been used for the benefit of researchers to push their professional or academic agendas forward, and not for the direct benefit of participants themselves [38]. In particular, issues surrounding trust may be exacerbated if refugees have a history of trauma, privacy concerns and undetermined status [39]. CBPR methodology attempts to address this by emphasizing the importance of ethical conduct of research within partner communities. Refugee partners also have important insights into how to better establish trusting relationships with their broader community, as demonstrated in the study by Goodkind et al. In order to create true, meaningful research partnerships with refugees, issues surrounding trust need to be addressed throughout the entire CBPR process, regardless of research stage.

Limitations to this study include the wide heterogeneity in study populations, methods and sample sizes, resulting in some difficulty in drawing conclusions from the literature. There is scarce literature describing recent and effective interventions and policies that have been informed by the involvement of refugees at every step of the CBPR process. As such, a scoping review methodology is indeed appropriate.

Based on the literature, key recommendations to increase the level of involvement and engagement of refugee communities are listed below and summarized in Table 3

1. **Improve participation accessibility in all stages of the research process:** In order to ensure more refugee partnerships, it is important that their involvement is made more accessible. This is needed in particular phases of the research process and must be enabled by those on the research team, as well as those at the funder/government agency level.
  - *At the researcher level:* Refugee participation is needed in step 2, obtaining funding. This will allow for 1) refugees to take ownership of their work, 2) support capacity building for refugee communities to initiate, lead and sustain projects within their communities, and 3) improve refugee autonomy, where they feel they are able to have a larger say in the research process if they are able to obtain funding for themselves. As funding is almost exclusively provided to academic centres, non-for-profit organizations, and other established research centres, it is important to ensure refugees are employed by these centres to truly establish ownership of their work.
  - *At the funder/government agency level:* This recommendation is two-fold. Firstly, funding bodies must put a greater emphasis on the importance of CBPR refugee community partners, as their involvement in research is key to creating sustainable and effective health interventions. Secondly, funding applications must be developed in a way that is accessible for refugees. Translated versions must be made available.
2. **Expand health intervention areas that have CBPR refugee involvement:** In this scoping review, we found that almost 79% of articles focused on interventions that addressed either mental health, social support or sexual and reproductive health. While these areas are of high priority, it is important that there is an expansion amongst the health topics supported by refugee partners. These may include involvement in the development of interventions that address chronic health conditions commonly reported amongst refugee communities, such as oral health, high cholesterol, anemia and high blood pressure [40, 41].
3. **Recognize and leverage refugee expertise within their communities:** Refugees have unique lived experience and have important insights into the contextual realities of their resettled communities. They understand the nuances in working within their communities that can truly transform effective research practices. They have successful ideas in terms of creating trust amongst community members, and their expertise needs to be more widely understood and emphasized. Efforts need to be made to empower and enable the community to take on leadership roles and vocalize their own lived experiences.
4. **Report data on race/racism, ethnicity and age independently:** It is well understood that one’s race and ethnicity are different entities and may impact an individual’s lived experiences differently [42]. In terms of race and racism, race/ethnicity are often reported (albeit the terms are usually conflated), but experiences of racism may not be explicitly explored. Race and ethnicity need to be reported separately as discussed previously, and experiences of racism must be further elucidated in future studies.

**Table 3:** Recommendations

**Recommendations**	
***Improve participation accessibility in all stages of the research process:*** refugee involvement must be made more accessible at the researcher level, and at the funder/government agency level.	
***Expand health intervention areas that have CBPR refugee involvement:*** it is important that there is an expansion amongst the health topics supported by refugee partners.	
***Recognize and leverage refugee expertise within their communities:*** Refugee communities have unique lived experience and have important insights into the contextual realities of their resettled communities. Efforts need to be made to empower and enable the community to take on leadership roles and vocalize their own lived experiences.	
***Report data on race/racism, ethnicity and age independently***	

## Conclusion

This scoping review is the first to systematically review published literature examining refugee involvement through CBPR processes to study and design healthcare interventions. It is evident that there are gaps pertaining to the extent of meaningful involvement of refugees in key stages of the research process, specifically in obtaining funding and scale up opportunities. This study highlights the need for dedicated efforts to increase the involvement of refugee communities as partners in research. Future research should aim to evaluate programs that have established refugee community partnerships to better understand their processes and outcomes. Studies should also explore the long-term impacts of having refugee communities as research partners in the populations they are working with, including the health outcomes of those populations. Future work may also include the use of refugee partners in policy design and implementation. As health intervention and policy research moves towards patient-oriented frameworks, it is also important to consider the barriers that prevent refugee communities and other marginalized groups from setting research priorities and engaging in the research process.

## Supplementary Information

**Additional file 1.** Search Strategies.

**Additional file 2.** PRISMA-S Checklist.

**Additional file 3.** Preferred Reporting Items for Systematic reviews and Meta-Analyses extension for Scoping Reviews (PRISMA-ScR) Checklist.

## Data Availability

All additional files and PRISMA checklists are included in this document.

## References

[1] Alencar A, Montes NM, Vicente-Mariño M (2019). From fragmentation to integration: addressing the role of communication in refugee crises and (re) settlement processes. Int Commun Gaz.

[2] Valipour M, Bateni SM, Jun C (2021). Global surface temperature: a new insight. Climate.

[3] Fazel M, Reed RV, Panter-Brick C, Stein A (2012). Mental health of displaced and refugee children resettled in high-income countries: risk and protective factors. Lancet.

[4] Kirmayer LJ, Narasiah L, Munoz M, Rashid M, Ryder AG, Guzder J, Hassan G, Rousseau C, Pottie K, for the Canadian Collaboration for Immigrant and Refugee Health (CCIRH) (2011). Common mental health problems in immigrants and refugees: general approach in primary care. CMAJ.

[5] Pauly B, Urbanoski K, Hartney E, Shahram S, Marcellus L, Wallace B, et al. What is missing from “patient oriented research”? A View from Public Health Systems and Services. Healthc Policy. 2019; https://www.longwoods.com/content/26075//what-is-missing-from-patient-oriented-research-a-view-from-public-health-systems-and-services (accessed 28 April 2021). 10.12927/hcpol.2019.26075 PMC7020799 32077841

[6] Matlin SA, Depoux A, Schütte S, Flahault A, Saso L (2018). Migrants’ and refugees’ health: towards an agenda of solutions. Public Health Rev.

[7] Betancourt TS, Frounfelker R, Mishra T, Hussein A, Falzarano R (2015). Addressing health disparities in the mental health of refugee children and adolescents through community-based participatory research: a study in 2 communities. Am J Public Health.

[8] Johnson CE, Ali SA, Shipp MP (2009). Building community-based participatory research partnerships with a Somali refugee community. Am J Prev Med.

[9] Burke JG, Hess S, Hoffmann K, Guizzetti L, Loy E, Gielen A, Bailey M, Walnoha A, Barbee G, Yonas M (2013). Translating community-based participatory research principles into practice. Prog Community Health Partners.

[10] Salazar LF, Crosby RA, DiClemente RJ (2015). Research methods in health promotion.

[11] Vaughn LM, Jacquez F, Lindquist-Grantz R, Parsons A, Melink K (2016). Immigrants as research partners: a review of immigrants in community-based participatory research (CBPR). J Immigr Minor Health.

[12] McGowan J, Sampson M, Salzwedel DM, Cogo E, Foerster V, Lefebvre C (2016). PRESS peer review of electronic search strategies: 2015 guideline statement. J Clin Epidemiol.

[13] United Nations High Commissioner for Refugees. Convention and Protocol Relating to the Status of Refugees: UNHCR. Geneva. https://www.unhcr.org/3b66c2aa10 12344222

[14] Arksey H, O’Malley L (2005). Scoping studies: towards a methodological framework. Int J Soc.

[15] Tricco AC, Lillie E, Zarin W, O'Brien KK, Colquhoun H, Levac D, Moher D, Peters MD, Horsley T, Weeks L, Hempel S (2018). PRISMA extension for scoping reviews (PRISMA-ScR): checklist and explanation. Ann Intern Med.

[16] Rethlefsen ML, Kirtley S, Waffenschmidt S, Ayala AP, Moher D, Page MJ, Koffel JB, PRISMA-S Group (2021). PRISMA-S: an extension to the PRISMA Statement for Reporting Literature Searches in Systematic Reviews. Syst Rev.

[17] Darroch F, Giles A (2014). Decolonizing Health Research: community-based participatory research and postcolonial feminist theory. Can J Action Res.

[18] Afifi RA, Makhoul J, El Hajj T, Nakkash RT (2011). Developing a logic model for youth mental health: participatory research with a refugee community in Beirut. Health Policy Plan.

[19] Baird MB, Domian EW, Mulcahy ER, Mabior R, Jemutai-Tanui G, Filippi MK (2015). Creating a Bridge of Understanding between Two Worlds: Community-Based Collaborative-Action Research with Sudanese Refugee Women. Public Health Nurs.

[20] Betancourt TS, Berent JM, Freeman J, Frounfelker RL, Brennan RT, Abdi S, Maalim A, Abdi A, Mishra T, Gautam B, Creswell JW, Beardslee WR (2020). Family-Based Mental Health Promotion for Somali Bantu and Bhutanese Refugees: Feasibility and Acceptability Trial. J Adolesc Health.

[21] Goodkind JR, Amer S, Christian C, Hess JM, Bybee D, Isakson BL, Baca B, Ndayisenga M, Greene RN, Shantzek C (2017). Challenges and innovations in a community-based participatory randomized controlled trial. Health Educ Behav.

[22] Grigg-Saito D, Och S, Liang S, Toof R, Silka L (2008). Building on the strengths of a Cambodian refugee community through community-based outreach. Health Promot Pract.

[23] Guerin PB, Allotey P, Hussein Elmi F, Baho S (2006). Advocacy as a means to an end: assisting refugee women to take control of their reproductive health needs. Women Health.

[24] Gustafson DT, Iluebbey V (2013). “traditional discipline” or domestic violence: participatory action research with a Sudanese refugee community. J Cult Divers.

[25] Miller KE, Ghalayini H, Arnous M, Tossyeh F, Chen A, van den Broek M, Koppenol-Gonzalez GV, Saade J, Jordans MJD (2020). Strengthening parenting in conflict-affected communities: development of the Caregiver Support Intervention. Glob Ment Health (Camb).

[26] Pavlish C, Ateva E, Ho A (2017). Women’s health and human rights: converging avenues for action in East Africa. J Hum Rights Pract.

[27] Riggs E, Muyeen S, Brown S, Dawson W, Petschel P, Tardiff W, Norman F, Vanpraag D, Szwarc J, Yelland J (2017). Cultural safety and belonging for refugee background women attending group pregnancy care: an Australian qualitative study. Birth.

[28] Signorelli RG, Coello M, Momartin S. Change and recovery: culturally appropriate early childhood Programmes with refugee families and communities. Child Aust Cambridge Univ Press. 2015;40(3):195–204. 10.1017/cha.2015.29.

[29] Stewart M, Simich L, Beiser M, Makumbe K, Makwarimba E, Shizha E (2011). Impacts of a social support intervention for Somali and Sudanese refugees in Canada. Ethn Inequal Health Soc Care.

[30] Tanabe M, Pearce E, Krause SK (2018). “Nothing about us, without us”: conducting participatory action research among and with persons with disabilities in humanitarian settings. Action Res.

[31] Warner G, Baghdasaryan Z, Osman F, Lampa E, Sarkadi A. ‘I felt like a human being’-An exploratory, multi-method study of refugee involvement in the development of mental health intervention research. Health Expect. 2019;24(S1):30–39. 10.1111/hex.12990 PMC8137488 31705620

[32] Israel BA, Coombe CM, Cheezum RR, Schulz AJ, Mcgranaghan RJ, Lichtenstein R (2010). Community-based participatory research: a capacity-building approach for policy advocacy aimed at eliminating health disparities. Am J Public Health.

[33] Wallerstein N. Commentary on Community-Based Participatory Research and Community Engaged Research in Health for Journal of Participatory Research Methods. J Particip Res Methods. 2020. 10.35844/001c.13274.

[34] Ford ME, Kelly PA (2005). Conceptualizing and categorizing race and ethnicity in health services research. Health Serv Res.

[35] Smedley A, Smedley B (2005). Race as biology is fiction, racism as a social problem is real. Am Psychol.

[36] Johnston-Guerrero MP (2016). Embracing the messiness: critical and diverse perspectives on racial and ethnic identity development. New Dir Stud Serv.

[37] Raghallaigh MI (2014). The causes of mistrust amongst asylum seekers and refugees: insights from research with unaccompanied asylum-seeking minors living in the Republic of Ireland. J Refug Stud.

[38] Dyregrov K, Dyregrov A, Raundalen M (2000). Refugee Families' experience of research participation. J Trauma Stress.

[39] Enticott JC, Shawyer F, Vasi S, Buck K, Cheng I, Russell G, Kakuma R, Minas H, Meadows G (2017). A systematic review of studies with a representative sample of refugees and asylum seekers living in the community for participation in mental health research. BMC Med Res Methodol.

[40] Lane G, Farag M, White J, Nisbet C, Vatanparast H (2018). Chronic health disparities among refugee and immigrant children in Canada. Appl Physiol Nutr Metab.

[41] Redditt VJ, Graziano D, Janakiram P, Rashid M (2015). Health status of newly arrived refugees in Toronto, Ont: part 2: chronic diseases. Can Fam Physician.

[42] Suleman S, Garber KD, Rutkow L (2018). Xenophobia as a determinant of health: an integrative review. J Public Health Pol.

