# Supplementary material for: A chair at the table: a scoping review of the participation of refugees in community-based participatory research in healthcare
Source: Global Health. 2021 Sep 6;17:103. doi: 10.1186/s12992-021-00756-7 (PMC8420006; doi:10.1186/s12992-021-00756-7)
Supplement: Supplementary file 1 — Additional file 1. Search Strategies. [file 12992_2021_756_MOESM1_ESM.docx]

**Additional file 1.** Search Strategies

Medline

Database(s): **Ovid MEDLINE: Epub Ahead of Print, In-Process & Other Non-Indexed Citations, Ovid MEDLINE® Daily and Ovid MEDLINE®** 1946-Present

| **#** | **Searches** | **Results** |
| --- | --- | --- |
| 1 | exp Community-Based Participatory Research/ | 4505 |
| 2 | Community Participation/ | 17180 |
| 3 | CBPR.ti,ab. | 1080 |
| 4 | ((Action or participatory or collaborative) adj2 research).ab,kf,ti. | 13503 |
| 5 | ((community-based or community-partnered or consumer-driven) adj3 research).ab,kf,ti. | 4436 |
| 6 | ((mutual or cooperative or co-operative or collaborative) adj2 inquiry).ab,kf,ti. | 143 |
| 7 | ((public or community or consumer) adj1 (participation or involvement* or engagement*)).ab,kf,ti. | 11522 |
| 8 | (participatory evaluation or action science or action* community or empowerment evaluation or participatory rural appraisal or active involvement or active collaboration or participatory approach).ab,kf,ti. | 3477 |
| 9 | ((policymak* or policy-mak* or policy development or policy design or decision-maker* or decisionmaker* or decision maker* or stakeholder* or stakeholder* or stake holder* or advisor* or consult* or active*) adj3 (participate* or involve* or engage* or role)).ab,kf,ti. | 40065 |
| 10 | or/1-9 | 80800 |
| 11 | Refugees/ | 10232 |
| 12 | Refugee Camps/ | 152 |
| 13 | (refugee* or displaced person* or displaced people or war prisoner* or undocumented migrant* or asylum* or transient* or displacement or forced migration* or host countr* or POW or stateless* or migrant* or sanctuary).ab,kf,ti. | 457064 |
| 14 | "Transients and Migrants"/ | 11652 |
| 15 | Prisoners of War/ | 509 |
| 16 | 11 or 12 or 13 or 14 or 15 | 463080 |
| 17 | 10 and 16 | 1343 |
| 18 | limit 17 to english language | 1298 |
| 19 | (review or systematic review).pt. | 2714752 |
| 20 | (systematic review or scoping review).m_titl. | 130723 |
| 21 | 19 or 20 | 2740306 |
| 22 | 18 not 21 | 1175 |

**PubMed**

(((("Community-Based Participatory Research"[Mesh] OR "Community Participation"[Mesh]) OR (("action research"[Title/Abstract] OR "participatory research"[Title/Abstract] OR "collaborative research"[Title/Abstract] OR "community-based research"[Title/Abstract] OR "community-partnered research"[Title/Abstract] OR "cooperative inquiry"[Title/Abstract] OR "co-operative inquiry"[Title/Abstract] OR "collaborative inquiry"[Title/Abstract] OR "Community-Based Participatory"[Title/Abstract] OR "participatory evaluation"[Title/Abstract] OR "empowerment evaluation"[Title/Abstract] OR "participatory rural appraisal"[Title/Abstract] OR "active involvement"[Title/Abstract] OR "participatory approach"[Title/Abstract]) OR ("action research"[Other Term] OR "participatory research"[Other Term] OR "collaborative research"[Other Term] OR "community-based research"[Other Term] OR "community-partnered research"[Other Term] OR "cooperative inquiry"[Other Term] OR "co-operative inquiry"[Other Term] OR "collaborative inquiry"[Other Term] OR "Community-Based Participatory"[Other Term] OR "participatory evaluation"[Other Term] OR "empowerment evaluation"[Other Term] OR "participatory rural appraisal"[Other Term] OR "active involvement"[Other Term] OR "participatory approach"[Other Term]))) OR ((policymak*[Title/Abstract] OR policy-mak*[Title/Abstract] OR policy development[Title/Abstract] OR policy design[Title/Abstract] OR decision-maker*[Title/Abstract] OR decisionmaker*[Title/Abstract] OR decision maker*[Title/Abstract] OR advisor*[Title/Abstract] OR consult*[Title/Abstract]) AND (participate*[Title/Abstract] OR involve*[Title/Abstract] OR engage*[Title/Abstract] OR role[Title/Abstract]))) AND (("Refugees"[Mesh] OR "Refugee Camps"[Mesh] OR "Transients and Migrants"[Mesh] OR "Prisoners of War"[Mesh]) OR (refugee*[Title/Abstract] OR "displaced person*"[Title/Abstract] OR "displaced people"[Title/Abstract] OR "war prisoner*"[Title/Abstract] OR "undocumented migrant*"[Title/Abstract] OR asylum*[Title/Abstract] OR transient*[Title/Abstract] OR displacement[Title/Abstract] OR "forced migration*"[Title/Abstract] OR "host countr*"[Title/Abstract] OR POW[Title/Abstract] OR sanctuary[Title/Abstract]))) NOT ((review[Publication Type] OR systematic review[Publication Type]) OR (systematic review[Title] OR scoping review[Title]))

EMBASE

Database(s): **Embase Classic+Embase** 1947 to 2020 August 05

| **#** | **Searches** | **Results** |
| --- | --- | --- |
| 1 | exp participatory research/ | 5549 |
| 2 | exp community participation/ | 2482 |
| 3 | ((Action or participatory or collaborative) adj2 research).ab,hw,ti. | 18602 |
| 4 | CBPR.ab,ti. | 1230 |
| 5 | ((community-based or community-partnered or consumer-driven) adj3 research).ab,hw,ti. | 4412 |
| 6 | ((mutual or cooperative or co-operative or collaborative) adj2 inquiry).ab,hw,ti. | 159 |
| 7 | ((public or community or consumer) adj1 (participation or involvement* or engagement*)).ab,hw,ti. | 14161 |
| 8 | (participatory evaluation or action science or action* community or empowerment evaluation or participatory rural appraisal or active involvement or active collaboration or participatory approach).ab,hw,ti. | 4403 |
| 9 | ((policymak* or policy-mak* or policy development or policy design or decision-maker* or decisionmaker* or decision maker* or stakeholder* or stakeholder* or stake holder* or advisor* or consult* or active*) adj3 (participate* or involve* or engage* or role)).ab,hw,ti. | 55074 |
| 10 | or/1-9 | 88165 |
| 11 | exp refugee/ | 13425 |
| 12 | refugee camp/ | 690 |
| 13 | (refugee* or displaced person* or displaced people or war prisoner* or undocumented migrant* or asylum* or transient* or displacement or forced migration* or host countr* or POW or stateless* or migrant or migrants or sanctuary).ab,hw,ti. | 608071 |
| 14 | "prisoner of war"/ | 461 |
| 15 | 11 or 12 or 13 or 14 | 608468 |
| 16 | 10 and 15 | 1642 |
| 17 | limit 16 to english language | 1595 |
| 18 | (books or chapter or conference abstract or conference paper or conference review or review).pt. | 7289628 |
| 19 | (systematic review or scoping review).m_titl. | 155732 |
| 20 | 18 or 19 | 7343720 |
| 21 | 17 not 20 | 1165 |
| 22 | remove duplicates from 21 | 1143 |

PsycInfo

Database(s): **APA PsycInfo** 1806 to July Week 4 2020

| **#** | **Searches** | **Results** |
| --- | --- | --- |
| 1 | community involvement/ | 4878 |
| 2 | action research/ or "Client Participation"/ | 5022 |
| 3 | exp focus group/ | 882 |
| 4 | ((Action or participatory or collaborative) adj2 research).ab,id,ti. | 14351 |
| 5 | CBPR.ab,ti. | 642 |
| 6 | ((community-based or community-partnered or consumer-driven) adj3 research).ab,id,ti. | 2887 |
| 7 | ((mutual or cooperative or co-operative or collaborative) adj2 inquiry).ab,id,ti. | 648 |
| 8 | ((public or community or consumer) adj1 (participation or involvement* or engagement*)).ab,id,ti. | 7467 |
| 9 | (participatory evaluation or action science or action* community or empowerment evaluation or participatory rural appraisal or active involvement or active collaboration or participatory approach).ab,id,ti. | 2414 |
| 10 | ((policymak* or policy-mak* or policy development or policy design or decision-maker* or decisionmaker* or decision maker* or stakeholder* or stakeholder* or stake holder* or advisor* or consult* or active*) adj3 (participate* or involve* or engage* or role or contribut*)).ab,id,ti. | 20837 |
| 11 | or/1-10 | 49774 |
| 12 | refugees/ | 6181 |
| 13 | political asylum/ | 36 |
| 14 | "prisoners of war"/ | 509 |
| 15 | (refugee* or displaced person* or displaced people or war prisoner* or undocumented migrant* or asylum* or transient* or displacement or forced migration* or host countr* or POW or stateless or migrant* or sanctuary).ab,id,ti. | 57496 |
| 16 | 12 or 13 or 14 or 15 | 57991 |
| 17 | 11 and 16 | 687 |
| 18 | limit 17 to english language | 646 |
| 19 | limit 18 to ("0100 journal" or "0110 peer-reviewed journal" or "0120 non-peer-reviewed journal" or "0130 peer-reviewed status unknown") | 485 |
| 20 | limit 18 to ("column/opinion" or "comment/reply" or editorial or "erratum/correction" or journal article or letter or retraction) | 467 |
| 21 | 19 or 20 | 485 |
| 22 | exp Health/ or exp Health Care Delivery/ or exp Health Care Services/ or exp Health Care Utilization/ or exp Primary Health Care/ | 429465 |
| 23 | exp Government Policy Making/ or exp Mental Health Services/ or exp Health Care Policy/ or exp Health Care Services/ or exp Public Health/ or exp Mental Health/ | 334906 |
| 24 | (health* or medical or clinical or dental or hospital or hospitalization or hospitalisation or clinic or medication*).ab,id,ti. | 1305822 |
| 25 | 22 or 23 or 24 | 1423760 |
| 26 | 21 and 25 | 244 |

**Scopus**

((TITLE-ABS(((Action or participatory or collaborative) W/2 research))) OR (TITLE-ABS(((community-based or community-partnered or consumer-driven) W/3 research))) OR (TITLE-ABS(((mutual or cooperative or co-operative or collaborative) W/2 inquiry))) OR (TITLE-ABS(((public or community or consumer) W/1 (participation or involvement* or engagement*)))) OR (TITLE-ABS("participatory evaluation" or "action science" or "action* community" or "empowerment evaluation" or "participatory rural appraisal" or "active involvement" or "active collaboration" or "participatory approach")) OR (TITLE-ABS(((policymak* or policy-mak* or "policy development" or "policy design" or decision-maker* or decisionmaker* or "decision maker*" or advisor* or consult* or active*) W/3 (participate* or involve* or engage* or role))))) AND (TITLE-ABS(refugee* or "displaced person*" or "displaced people" or "war prisoner*" or "undocumented migrant*" or asylum* or "forced migration*" or "host countr*" or POW or stateless or sanctuary)) AND (TITLE-ABS(health* or medical or clinical or dental or hospital or hospitalization or hospitalisation or clinic or medication*)) AND ( LIMIT-TO ( DOCTYPE,"ar" ) OR LIMIT-TO ( DOCTYPE,"ed" ) OR LIMIT-TO ( DOCTYPE,"er" ) OR LIMIT-TO ( DOCTYPE,"no" ) OR LIMIT-TO ( DOCTYPE,"Undefined" ) ) AND ( LIMIT-TO ( LANGUAGE,"English" ) )

**CINAHL**

| **#** | **Query** | **Limiters/Expanders** | **Last Run Via** | **Results** |
| --- | --- | --- | --- | --- |
| S12 | S8 AND S11 | Limiters - English Language; Publication Type: Abstract, Brief Item, Case Study, Commentary, Consumer/Patient Teaching Materials, Corrected Article, Editorial, Journal Article, Legal Case, Letter, Practice Guidelines, Questionnaire/Scale, Research, Response, Standards  Expanders - Apply equivalent subjects  Search modes - Boolean/Phrase | Interface - EBSCOhost Research Databases  Search Screen - Advanced Search  Database - CINAHL Plus with Full Text | 406 |
| S11 | S9 OR S10 | Expanders - Apply equivalent subjects  Search modes - Boolean/Phrase | Interface - EBSCOhost Research Databases  Search Screen - Advanced Search  Database - CINAHL Plus with Full Text | 56,311 |
| S10 | TI ( refugee* or displaced person* or displaced people or war prisoner* or undocumented migrant* or asylum* or transient* or displacement or forced migration* or host countr* or POW or stateless or migrant* or sanctuary ) OR AB ( refugee* or displaced person* or displaced people or war prisoner* or undocumented migrant* or asylum* or transient* or displacement or forced migration* or host countr* or POW or stateless or migrant* or sanctuary ) | Expanders - Apply equivalent subjects  Search modes - Boolean/Phrase | Interface - EBSCOhost Research Databases  Search Screen - Advanced Search  Database - CINAHL Plus with Full Text | 54,360 |
| S9 | (MH "Refugees") OR (MH "Refugee Camps") | Expanders - Apply equivalent subjects  Search modes - Boolean/Phrase | Interface - EBSCOhost Research Databases  Search Screen - Advanced Search  Database - CINAHL Plus with Full Text | 7,331 |
| S8 | S1 OR S2 OR S3 OR S4 OR S5 OR S6 OR S7 | Expanders - Apply equivalent subjects  Search modes - Boolean/Phrase | Interface - EBSCOhost Research Databases  Search Screen - Advanced Search  Database - CINAHL Plus with Full Text | 34,074 |
| S7 | TI ( ((policymak* or policy-mak* or policy development or policy design or decision-maker* or decisionmaker* or decision maker* or advisor* or consult* or active*) N3 (participate* or involve* or engage* or role or contribut*)) ) OR AB ( ((policymak* or policy-mak* or policy development or policy design or decision-maker* or decisionmaker* or decision maker* or advisor* or consult* or active*) N3 (participate* or involve* or engage* or role or contribut*)) ) | Expanders - Apply equivalent subjects  Search modes - Boolean/Phrase | Interface - EBSCOhost Research Databases  Search Screen - Advanced Search  Database - CINAHL Plus with Full Text | 13,355 |
| S6 | TI ( participatory evaluation or action science or action* community or empowerment evaluation or participatory rural appraisal or active involvement or active collaboration or participatory approach ) OR AB ( participatory evaluation or action science or action* community or empowerment evaluation or participatory rural appraisal or active involvement or active collaboration or participatory approach ) | Expanders - Apply equivalent subjects  Search modes - Boolean/Phrase | Interface - EBSCOhost Research Databases  Search Screen - Advanced Search  Database - CINAHL Plus with Full Text | 1,885 |
| S5 | TI ( ((public or community or consumer) N1 (participation or involvement* or engagement*)) ) OR AB ( ((public or community or consumer) N1 (participation or involvement* or engagement*)) ) | Expanders - Apply equivalent subjects  Search modes - Boolean/Phrase | Interface - EBSCOhost Research Databases  Search Screen - Advanced Search  Database - CINAHL Plus with Full Text | 7,376 |
| S4 | TI ( ((mutual or cooperative or co-operative or collaborative) N2 inquiry) ) OR AB ( ((mutual or cooperative or co-operative or collaborative) N2 inquiry) ) | Expanders - Apply equivalent subjects  Search modes - Boolean/Phrase | Interface - EBSCOhost Research Databases  Search Screen - Advanced Search  Database - CINAHL Plus with Full Text | 215 |
| S3 | TI ( ((community-based or community-partnered or consumer-driven) N3 research) ) OR AB ( ((community-based or community-partnered or consumer-driven) N3 research) ) | Expanders - Apply equivalent subjects  Search modes - Boolean/Phrase | Interface - EBSCOhost Research Databases  Search Screen - Advanced Search  Database - CINAHL Plus with Full Text | 2,535 |
| S2 | TI ( ((Action or participatory or collaborative) N2 research) ) OR AB ( ((Action or participatory or collaborative) N2 research) ) | Expanders - Apply equivalent subjects  Search modes - Boolean/Phrase | Interface - EBSCOhost Research Databases  Search Screen - Advanced Search  Database - CINAHL Plus with Full Text | 9,897 |
| S1 | (MH "Action Research") | Expanders - Apply equivalent subjects  Search modes - Boolean/Phrase | Interface - EBSCOhost Research Databases  Search Screen - Advanced Search  Database - CINAHL Plus with Full Text | 6,397 |

Database(s): **Global Health** 1973 to 2020 Week 30

| **#** | **Searches** | **Results** |
| --- | --- | --- |
| 1 | community involvement/ or community action/ or social participation/ | 5549 |
| 2 | ((Action or participatory or collaborative) adj2 research).ab,hw,ti. | 3597 |
| 3 | ((community-based or community-partnered or consumer-driven) adj3 research).ab,hw,ti. | 1694 |
| 4 | ((mutual or cooperative or co-operative or collaborative) adj2 inquiry).ab,hw,ti. | 15 |
| 5 | ((public or community or consumer) adj1 (participation or involvement* or engagement*)).ab,hw,ti. | 6952 |
| 6 | (participatory evaluation or action science or action* community or empowerment evaluation or participatory rural appraisal or active involvement or active collaboration or participatory approach).ab,hw,ti. | 1089 |
| 7 | ((policymak* or policy-mak* or policy development or policy design or decision-maker* or decisionmaker* or decision maker* or advisor* or consult* or active*) adj2 (participate* or involve* or engage* or role)).ab,hw,ti. | 3776 |
| 8 | or/1-7 | 15722 |
| 9 | refugees/ | 3473 |
| 10 | (refugee* or displaced person* or displaced people or war prisoner* or undocumented migrant* or asylum* or forced migration* or host countr* or POW or stateless or sanctuary).ab,hw,ti. | 6062 |
| 11 | 9 or 10 | 6062 |
| 12 | 8 and 11 | 166 |
| 13 | limit 12 to (english language and (annual report or annual report section or editorial or journal article or journal issue or standard or miscellaneous)) | 157 |

Policy File Index

| **Set#** | **Searched for** | **Databases** | **Results** |  |
| --- | --- | --- | --- | --- |
| S1 | (( action OR participatory OR collaborative ) P/2 research ) OR (( community-based OR community-partnered OR consumer-driven ) P/3 research ) OR (( mutual OR cooperative OR co-operative OR collaborative ) P/2 inquiry ) | Policy File Index | 135 | |
| S4 | (refugee* or displaced person* or displaced people or war prisoner* or undocumented migrant* or asylum* or forced migration* or host countr* or POW or stateless or sanctuary) | Policy File Index | 3247 | |
| S6 | (health* or medical or clinical or dental or hospital or hospitalization or hospitalisation or clinic or medication*) | Policy File Index | 43906 | |
| S8 | participatory evaluation or action science or action* community or empowerment evaluation or participatory rural appraisal or active involvement or active collaboration or participatory approach | Policy File Index | 2772 | |
| S10 | policymak* OR policy mak* OR policy development OR policy design OR decision maker* OR decisionmaker* OR decision maker* OR advisor* OR consult* OR active* | Policy File Index | 82136 | |
| S11 | S1 OR S8 OR S10 | Policy File Index  *These databases are searched for part of your query.* | 83351 | |
| S12 | S4 AND S6 AND S11 | Policy File Index  *These databases are searched for part of your query.* | 160 | |
| S13 | (S4 AND S6 AND S11) AND (subt.exact("health care policy" OR "foreign policy") AND la.exact("ENG")) | Policy File Index  *These databases are searched for part of your query.* | 51 | |
